# Bacterial Vaginosis and Trichomoniasis: Epidemiology and Management of Recurrent Disease

**DOI:** 10.1155/S1064744995000111

**Published:** 1995

**Authors:** David E. Soper

**Affiliations:** Department of Obstetrics and Gynecology Medical College of Virginia Virginia Commonwealth University Box 34, MCV Station Richmond VA 23298-0034 USA

## Abstract

Effective therapies exist for the treatment of both vaginal trichomoniasis and bacterial vaginosis (BV). Recurrent trichomonas infection is uncommon, and significant metronidazole resistance remains rare. The management of metronidazole-resistant trichomoniasis is dependent on susceptibility studies, which can be used to guide higher doses of metronidazole therapy. Recurrent BV is common. A mechanism for reestablishing the normal vaginal flora with H_2_O_2_-producing lactobacilli remains elusive. The management of this recurrent infection is based upon a longer duration of therapy with currently available antibiotic regimens and documentation of a clinical response using composite clinical criteria and Gram's stain of the vaginal secretions.


Infectious Diseases in Obstetrics and Gynecology 2:242–247 (1995)
© 1995 Wiley-Liss, Inc.

### Bacterial Vaginosis and Trichomoniasis: Epidemiology and Management of Recurrent Disease

**David E. Soper**

*Department of Obstetrics and Gynecology, Medical College of Virginia, Virginia Commonwealth University, Richmond, VA*

ABSTRACT

**Effective therapies exist for the treatment of both vaginal trichomoniasis and bacterial vaginosis (BV). Recurrent trichomonas infection is uncommon, and significant metronidazole resistance remains rare. The management of metronidazole-resistant trichomoniasis is dependent on susceptibility studies, which can be used to guide higher doses of metronidazole therapy. Recurrent BV is common. A mechanism for reestablishing the normal vaginal flora with $H_2O_2$-producing lactobacilli remains elusive. The management of this recurrent infection is based upon a longer duration of therapy with currently available antibiotic regimens and documentation of a clinical response using composite clinical criteria and Gram's stain of the vaginal secretions.** © 1995 Wiley-Liss, Inc.

KEY WORDS

Metronidazole, clindamycin, vaginitis

Vaginitis is one of the most common problems in clinical medicine, accounting for more than 10 million office visits each year. It is the most common reason for a patient to visit her obstetrician-gynecologist.[1] Bacterial vaginosis (BV) and vaginal trichomoniasis are 2 of the most common causes of infective vaginitis. While effective therapy for these diseases exists, recurrent disease is not uncommon. Crucial management strategies for patients with recurrent disease include confirmation of the diagnosis and clinical monitoring of the disorder while the patient undergoes antimicrobial therapy.

### RECURRENT TRICHOMONIASIS

*Trichomonas vaginalis* is one of the most common organisms causing vaginitis in women worldwide and affects approximately 3 million American women annually.[2] Metronidazole remains the drug of choice for the treatment of trichomonas vaginitis. The most extensively studied metronidazole regimens have been 200–250 mg t.i.d. for 7 days and a single 2-g dose; these regimens have median cure rates of 92% and 96%, respectively.[3] Treatment failures have been attributed to noncompliance with therapy, reinfection, or in situ inactivation or malabsorption of the drug.[4–6] Although metronidazole resistance is increasing, it is still uncommon.[7]

The 2-g single-dose regimen minimizes noncompliance and is more practical for the treatment of sexual partners. Sexual partners should be treated since reinfection rates of 6.2% to 23.7% are noted in women whose sexual partners are not treated simultaneously.[6–8] In addition, *T. vaginalis* can be a cause of tetracycline-resistant nongonococcal urethritis in males.

### Recommended Regimens

Metronidazole is the only trichomonacidal drug available in the United States. Metronidazole, 2 g orally in a single dose, is the current recommended regimen for the treatment of both men and women with trichomoniasis.[9] An alternative regimen is

**Address correspondence/reprint requests to Dr. David Soper, Department of Obstetrics and Gynecology, Medical College of Virginia, Virginia Commonwealth University, Box 34, MCV Station, Richmond, VA 23298-0034.**

Received April 26, 1994

Review Article

Accepted August 9, 1994

**TABLE 1. Treatment of metronidazole-resistant trichomoniasis[a]**

| Resistance level | Aerobic MLC (μg/ml) | Metronidazole regimen |
|---|---|---|
| Marginal | 50 | Retreatment with standard dose |
| Low | <100 | 2 g qd for 3–5 days |
| Moderate | 100–200 | 2–2.5 g qd for 7–10 days |
| High | ≥200 | 3–3.5 g qd for 14–18 days[b] |
| High (alternate) | | 2 g IV q 6–8 h for 3 days |

[a]Modified from references 3, 5, and 11.
[b]Concomitant vaginal treatment may be helpful.

metronidazole, 500 mg orally b.i.d. for 7 days. If failure occurs with either regimen, the patient should be retreated with one of these standard regimens. Eighty-five percent of initial treatment failures will respond to a repeat single 2-g dose of metronidazole.[10] If repeated failure occurs, the patient should be treated with a 2-g dose of metronidazole daily for 3–5 days. Compliance issues and the possibility of reinfection should be addressed. Cases of additional culture-documented treatment failure should be managed in consultation with an expert. The evaluation of such cases should include a determination of the susceptibility of *T. vaginalis* to metronidazole.

### Metronidazole Resistance

In 1979, reports concerning the emergence of metronidazole-resistant strains of *T. vaginalis* were published.[11–13] Similar cases became clinically evident in the United States and prompted the Centers for Disease Control (CDC) to evaluate the susceptibility to metronidazole of isolates from women failing at least 2 standard treatment regimens with metronidazole and in whom reinfection was unlikely because of the interim absence of coitus.[14] Although methods for determining the sensitivity of *T. vaginalis* are not standardized, the testing procedures identify 4 levels of metronidazole resistance: marginal, low, moderate, and high (Table 1). Aerobic values for the mean lethal concentration (MLC), or trichomonacidal level of metronidazole, appear to correlate better with the clinical response to therapy than the results of susceptibility testing done under anaerobic conditions. Although there is considerable overlap in the MLC values of isolates from responsive and unresponsive infections, an aerobic MLC value of >100 μg/ml is highly associated with clinical resistance.[15]

Patients with marginally resistant organisms respond well to repeat treatment with standard doses. Increased resistance requires larger doses of metronidazole administered for longer periods (Table 1).

As noted above, patients with repeated treatment failure should have cultures of their vaginal secretions and the *T. vaginalis* isolate tested for its susceptibility. This technique involves suspending a swab of the vaginal secretions in modified Diamond's medium followed by incubation at 35–37°C for ≥24 h. This culture medium should be prewarmed prior to inoculating the vaginal sample. Once a positive culture is confirmed by a wet preparation of the original culture, a second prewarmed culture tube should be inoculated and mailed by overnight delivery service to a reference laboratory capable of performing susceptibility testing. The patient may be placed on intravaginal clotrimazole in an attempt to ameliorate her symptoms while awaiting the results of the *T. vaginalis* susceptibility testing.

Once the level of resistance is known, appropriate doses of metronidazole should be prescribed (Table 1). Concurrent treatment with vaginal metronidazole has been recommended for patients with isolates showing a high level of resistance. Prior to the availability of 0.75% metronidazole vaginal gel, 500-mg tablets of metronidazole (not enteric coated) were used as vaginal suppositories.[4] Now, 5 g of gel (37.5 mg of metronidazole) may be used intravaginally b.i.d. as an adjunct to high-dose oral therapy in highly resistant cases.

Patients with significant resistance should be evaluated for parasite persistence with a wet preparation of the vaginal secretions prior to discontinuing metronidazole therapy. If motile trichomonads are noted, continued therapy is indicated or consideration of higher doses of metronidazole. Once the parasite disappears from the wet preparation, a culture should be performed to ensure that low levels of trichomonads do not remain. A follow-up appointment 6 weeks after therapy has been completed is also recommended. A repeat evaluation of the vaginal secretions and trichomonas culture should be performed at this time.

Oral absorption of metronidazole is almost complete. The drug is metabolized in the liver, and the hydroxy metabolite may act synergistically with the parent compound. Nausea and vomiting develop less frequently during IV than oral therapy at normal-to-moderate doses. However, high doses may lead to gastrointestinal side effects regardless of the route of administration and may be due to the accumulation of the hydroxy metabolite. For this reason, hospitalization for the parenteral use of metronidazole for the treatment of resistant trichomonas vaginitis is usually not recommended. Other manifestations of metronidazole toxicity include metallic taste, glossitis, stomatitis, urticaria, vertigo, convulsive seizures, and peripheral neuropathy. Neurotoxicity appears to be a consistent problem when high-dose treatment exceeds 3 or 4 days.[7]

A number of similar nitroimidazole derivatives, such as tinidazole and ornidazole, are available in countries worldwide including Canada and the Bahamas, but not the United States. Although cross resistance is common, it is often incomplete. These alternative agents can be considered if susceptibility studies suggest that certain metronidazole-resistant infections can be effectively treated with these antimicrobials.

Several investigators have reported the use of agents in addition to or other than metronidazole in the treatment of resistant trichomonal infections. Grossman and Galask[4] added a twice weekly 3% acetic acid vaginal wash to their metronidazole regimen in successfully treating 2 patients with metronidazole-resistant trichomoniasis. In a different case, a providone-iodine douche, 20 ml of a 10% solution, given b.i.d. for 4 days (8 douches) and repeated 2 weeks later for 2 days (4 douches) was successful in eradicating a metronidazole-resistant trichomonal infection that had previously been unresponsive to high-dose metronidazole and ornidazole.[16] Livengood and Lossick[17] reported that the routine use of a contraceptive suppository containing 100 mg of nonoxynol-9 resulted in complete resolution of vaginal irritative symptoms after the second episode of protected coitus. Further follow-up with wet preparations and culture documented resolution of the resistant trichomonas vaginitis. The prior use of gentian violet, nitrofurantoin, chlorhexidine, clotrimazole, povidone-iodine, 3% acetic acid, and hydrogen peroxide had been unsuccessful. Oral mebandazole with and without adjunctive vaginal mebendazole, although showing some in vitro activity against *T. vaginalis*, failed to eradicate the parasite in 2 women with resistant infection.[18] *Lactobacillus* immunotherapy using a vaccine of a lyophilisate of inactivated microorganisms of selected strains of *Lactobacillus acidophilus*, originally isolated from vaginal secretions of patients with trichomoniasis, was unsuccessfully used in conjunction with high-dose metronidazole in an attempt to eradicate a metronidazole-resistant trichomonal infection.[19]

### RECURRENT BV

BV is considered the most common vaginal infection in women. It is a complex alteration of the normal vaginal flora resulting in the replacement of a predominantly $H_2O_2$-producing lactobacilli melieu with a mixed flora of anaerobic bacteria, *Gardnerella vaginalis*, and *Mycoplasma hominis*. Common symptoms associated with BV include excessive vaginal discharge and vaginal malodor, although almost half of the women with this disorder are asymptomatic.[20] BV is associated with a number of infectious reproductive sequelae including upper genital tract infection, adverse pregnancy outcomes, and postoperative infection.[20] Of several antibiotics that have been studied, including sulfa vaginal creams, ampicillin, doxycycline, clindamycin, and metronidazole, the latter 2 appear to be the most effective. Clindamycin and metronidazole can be administered either orally or intravaginally for the treatment of BV.[21–27] These agents are associated with cure rates around 80% 4 weeks following treatment.

Long-term recurrence rates are observed in patients with BV irrespective of the treatment method. Sobel et al.[28] noted that over half of cured patients developed symptomatic clinical and laboratory mainfestations of BV within 3 months of completing therapy. Up to 80% of women developed recurrent BV within 9 months of therapy in a Seattle study.[29] The reasons for recurrence are not understood. One explanation suggests reinfection, either endogenously or by a male partner who is colonized with BV-associated microorganisms. Data indicating that the treatment of male sexual partners fails to reduce the incidence of recurrent BV argue against this latter theory.[30] Other causes for recurrence may be explained by the persistence of BV-associated microorganisms. This persistence may occur when the microorganisms are inhibited but not killed, and pathogenic bacteria gain predominance because the normal, protective lactobacillus-dominant flora has not been reestablished.[29,31]

Cook et al.,[31] in a study of 31 women with recurrent BV, noted that relapse appeared to reflect a failure of the complete normalization of the vaginal ecosystem without indicating resistance to metronidazole. Although the therapeutic implications of their observations remain conjectural, more prolonged antimicrobial therapy could conceivably result in a lower recurrence rate because of a diminished anaerobic flora. Because failure to reestablish a normal profile of lower-genital-tract flora is encountered, even among women considered clinically cured of BV, the restoration of a lactobacilli-dominant vaginal flora should be a central object of therapy. Intravaginal inoculation of *Lactobacillus* preparations could potentially improve outcome in these patients. Unfortunately, over-the-counter *Lactobacillus* preparations adhere poorly to vaginal epithelial cells, making vaginal colonization unlikely.[32] In addition, most commercially available *Lactobacillus* preparations do not contain the $H_2O_2$-producing strains known to be protective in the vagina.[33] Despite these observations, a diet associated with daily yogurt consumption has recently been shown to decrease the risk for recurrent vulvovaginal candidiasis.[34] Since the postulated mechanism of action concerns colonization resistance by lactobacilli of potential pathogens, a similar study should be performed with respect to women with recurrent BV.

### MANAGEMENT OF RECURRENT BV

The clinical approach to the management of women with symptomatic recurrent BV should first concern confirming the diagnosis. Composite clinical criteria (homogenous discharge, pH >4.5, clue cells, positive whiff test) should be used initially and confirmed by evaluating a Gram's stain of vaginal secretions from the lateral vaginal sidewall. Vulvovaginal candidiasis may be present in cases of recurrent BV and, if present, should be treated prior to or concurrently with the institution of therapy for BV.[35]

Once the diagnosis of BV is confirmed, treatment with an oral regimen of metronidazole or clindamycin should then be undertaken (Table 2). Although topical therapy is equally effective, it is crucial to be able to evaluate the vaginal secretions in women with recurrent or persistent symptoms to ensure that microscopy of a wet preparation confirms the normalization of the vaginal flora. Topical medications interfere with performing a wet preparation of the vaginal secretions; therefore, the oral route of administration is preferred. Therapy should be continued until an absence of clue cells is documented by microscopy and confirmed by a Gram's stain of vaginal secretions from the lateral vaginal sidewall. If, after 2 weeks of therapy, clue cells persist, a change to an alternative antibiotic is suggested. If both clindamycin and metronidazole are ineffective, this author has used amoxicillin/clavulanate with good results. Once the patient is rendered asymptomatic and a follow-up evaluation confirms the reestablishment of normal flora or at least an absence of clue cells, intermittent single-dose prophylaxis with 2 g of metronidazole once a month can be considered. The 2-g dose of metronidazole is the only regimen proved to be effective as single-dose therapy of BV.[22]

The microflora may not revert to a lactobacilli predominance during therapy or even immediately after therapy because of the activity of clindamycin or amoxicillin/clavulanate against lactobacilli. It sometimes takes several weeks for the lactobacilli predominance to appear in women after treatment and cure by clinical criteria.

**TABLE 2. Treatment of recurrent BV**

| Antimicrobial agent | Dose | Duration |
|---|---|---|
| Metronidazole | 500 mg p.o. b.i.d. | 14 days[a] |
| Clindamycin | 300 mg p.o. b.i.d. | 14 days[a] |
| Clindamycin | 2% vaginal cream qd | 14 days[a] |
| Metronidazole | 0.75% vaginal gel b.i.d. | 14 days[a] |
| Amoxicillin/clavulanate | 500 mg p.o. t.i.d. | 14 days[a] |
| Metronidazole | 2 g p.o. q month | 6 months[b] |

[a]Minimum duration of therapy. Discontinuation of antibiotic therapy is based upon normalization of the vaginal secretions as documented by microscopy of a wet preparation and Gram's stain.
[b]Prophylactic regimen.

The sexual transmission of BV is controversial.[36] Generally, treatment of the male sexual partner of a woman with BV is not recommended. However, BV-associated bacteria can be isolated from the urine and urethral scrapings of the male partners of women with BV. Moreover, treatment of both the patient and her partner for a longer time results in significantly higher cure rates.[37] For these reasons, concurrent therapy of the male sexual partner of the patient with *recurrent* BV may be considered. In addition, condom use should be recommended until such therapy is completed. Not only does condom use protect the patient from the potential of a sexually transmitted reinfection, but it also avoids alkalinization of the vagina by the ejaculate, which may help in reestablishing a normal vaginal pH and therefore normal vaginal flora.

### REFERENCES

1. Kent HL: Epidemiology of vaginitis. Am J Obstet Gynecol 165:1168–1176, 1991.
2. Rein MF: *Trichomonas vaginalis.* In Mandell GL, Douglas RG, Jr, Bennett JE (eds): Principles and Practice of Infectious Diseases. 3rd ed. New York: Churchill-Livingstone, pp 2115–2118, 1990.
3. Lossick JG: Treatment of sexually transmitted vaginosis/vaginitis. Rev Infect Dis 12(suppl):S665–681, 1990.
4. Grossman JH, Galask RP: Persistent vaginitis caused by metronidazole-resistant trichomonas. Obstet Gynecol 76:521–522, 1990.
5. Mead PB, Gibson M, Schentag JJ, Ziemniak JA: Possible alteration of metronidazole metabolism by phenobarbital [Letter]. N Engl J Med 306:1490, 1982.
6. Ahmed-Jushuf IH, Murray AE, McKeown J: Managing trichomonal vaginitis refractory to conventional treatment with metronidazole. Genitourin Med 64:25–29, 1988.
7. Lossick JG, Kent HL: Trichomoniasis: Trends in diagnosis and management. Am J Obstet Gynecol 165:1217–1222, 1991.
8. Sands RX: Pregnancy, trichomoniasis, and metronidazole: A novel dose schedule. Am J Obstet Gynecol 94:350–355, 1966.
9. Peterson WF, Stauch JE, Ryder CD: Metronidazole in pregnancy. Am J Obstet Gynecol 94:343–349, 1966.
10. Lyng J, Christensen J: A double-blind study of the value of treatment with a single dose of tinidazole to partners to females with trichomoniasis. Acta Obstet Gynaecol Scand 60:199–201, 1981.
11. CDC: 1993 Sexually transmitted diseases treatment guidelines. MMWR 42:70–72, 1993.
12. Muller M, Lossick JG, Gorrell TE: In vitro susceptibility of *Trichomonas vaginalis* to metronidazole and treatment outcome in vaginal trichomoniasis. Sex Transm Dis 15:17–24, 1988.
13. Forsgren A, Forssman L: Metronidazole-resistant *Trichomonas vaginalis.* Br J Vener Dis 55:351–353, 1979.
14. Meingassner JG, Thurner J: Strain of *Trichomonas vaginalis* resistant to metronidazole and other 5-nitroimidazoles. Antimicrob Agents Chemother 15:254–257, 1979.
15. Lossick JG, Muller M, Gorrell TE: In vitro susceptibility and doses of metronidazole required for cure in cases of refractory vaginal trichomoniasis. J Infect Dis 153:948–955, 1986.
16. Wong CA, Wilson PD, Chew TA: Povidone-iodine in the treatment of metronidazole-resistant *Trichomonas vaginalis.* Aust NZ J Obstet Gynecol 30:169–171, 1990.
17. Livengood CH, Lossick JG: Resolution of resistant vaginal trichomoniasis associated with the use of intravaginal nonoxynol-9. Obstet Gynecol 78:954–956, 1991.
18. Pattman, RS, Sprott MS, Kearns AM, Earnshaw M: Failure of mebendazole to cure trichomonal vaginitis resistant to metronidazole: Case reports. Genitourin Med 65:274–275, 1989.
19. van der Weiden RMF, van der Meijden WI, Bogchelman DH, Polderman AM: Treatment failure in trichomoniasis and persistance of the parasite after *Lactobacillus* immunotherapy: Two case reports. Eur J Obstet Gynaecol Reprod Biol 34:171–178, 1990.
20. Eschenbach DA, Hillier S, Critchlow, Stevens C, De Rousen T, Holmes KK: Diagnosis and clinical manifestations of bacterial vaginosis. Am J Obstet Gynecol 158:819–828, 1988.
21. Spiegal CA: Bacterial vaginosis. Clin Microbiol Rev 4:485–502, 1991.
22. Lugo-Miro VI, Green M, Mazur L: Comparison of different metronidazole therapeutic regimens for bacterial vaginosis. A meta-analysis. JAMA 268:92–95, 1992.
23. Schmitt C, Sobel JD, Meriwether C: Bacterial vaginosis: Treatment with clindamycin cream versus oral metronidazole. Obstet Gynecol 79:1020–1023, 1992.
24. Fischbach F, Petersen EE, Weissenbacher ER, Martius J, et al.: Efficacy of clindamycin vaginal cream versus oral metronidazole in the treatment of bacterial vaginosis. Obstet Gynecol 82:405–410, 1993.
25. Hillier SL, Lipinski C, Briselden AM, Eschenbach DA: Efficacy of intravaginal 0.75% metronidazole gel for the treatment of bacterial vaginosis. Obstet Gynecol 81:963–967, 1993.
26. Livengood CH III, McGregor JA, Soper DE, Newton E, Thomason JL: Bacterial vaginosis: Efficacy and safety of intravaginal metronidazole treatment. Am J. Obstet Gynecol 170:759–764, 1994.
27. Sweet RL: New approaches for the treatment of bacterial vaginosis. Am J Obstet Gynecol 169:479–482, 1993.
28. Sobel JD, Schmitt C, Meriwether C: Long-term follow-up of patients with bacterial vaginosis treated with oral metronidazole and topical clindamycin [Letter]. J Infect Dis 167:783–784, 1993.
29. Hillier S, Holmes KK: Bacterial vaginosis. In Holmes KK, Mardh P-A, Sparling PF (eds): Sexually Transmitted Diseases. 2nd ed. New York: McGraw-Hill, p 557, 1990.

30. Swedberg J, Steiner JF, Deiss F, et al.: Comparison of single dose vs. one-week course of metronidazole for symptomatic bacterial vaginosis. JAMA 254:1046–1049, 1985.
31. Cook RL, Redondo-Lopez V, Schmitt C, Meriwether C, Sobel JD: Clinical, moicrobiological, and biochemical factors in recurrent bacterial vaginosis. J Clin Microbiol 30:870–877, 1992.
32. Wood JR, et al.: In vitro and adherence of *Lactobacillus* species to vaginal epithelial cells. Am J Obstet Gynecol 153:740–743, 1985.
33. Hughes VL, Hillier SL: Lactobacilli from non-prescription products used for the treatment of vaginitis. Obstet Gynecol 75:244–248, 1990.
34. Hilton E, Isenberg HD, Alperstein P, France K Borenstein MT: Ingestion of yogurt containing *Lactobacillus acidophilus* as prophylaxis for candidal vaginitis. Ann Intern Med 116:353–357, 1992.
35. Redondo-Lopez V, Meriwether C, Schmitt, el al.: Vulvovaginal candidiasis complicating recurrent bacterial vaginosis. Sex Transm Dis 17:51–53, 1990.
36. Thomason JL, Gelbart SM, Scaglione NJ: Bacterial vaginosis: Current review with indications for asymptomatic therapy. Am J Obstet Gynecol 165:1210–1217, 1991.
37. Mengel MB, Berg AO, Weaver CH, et al.: The effectiveness of single-dose metronidazole therapy for patients and their partners with bacterial vaginosis. J Fam Pract 28:163–171, 1989.
